# Right ventricular hypertrophy after atrial switch operation: normal adaptation process or risk factor? A cardiac magnetic resonance study

**DOI:** 10.1007/s00392-012-0485-6

**Published:** 2012-06-20

**Authors:** Matthias Grothoff, Janine Hoffmann, Hashim Abdul-Khaliq, Lukas Lehmkuhl, Ingo Dähnert, Felix Berger, Meinhard Mende, Matthias Gutberlet

**Affiliations:** 1 Department of Diagnostic and Interventional Radiology, Heart Center, University of Leipzig, Strümpellstr. 39, 04289 Leipzig, Germany; 2 Department of Congenital Heart Disease/Pediatric Cardiology, University Saarland, Kirrberger Str. Gebäude 9, 66421 Homburg/Saar, Germany; 3 Department of Congenital Heart Disease/Pediatric Cardiology, Heart Center, University Leipzig, Strümpellstr. 39, 04289 Leipzig, Germany; 4 Department of Congenital Heart Disease/Pediatric Cardiology, Berlin Heart Center, Augustenburger Platz 1, 13353 Berlin, Germany; 5 Clinical Trial Centre, University of Leipzig, Leipzig, Germany; 6 Department of Obstetrics, University of Leipzig, Liebigstr. 20a, 04103 Leipzig, Germany

**Keywords:** Transposition of the great arteries, Systemic right ventricle, Cardiac magnetic resonance imaging, Right ventricular failure, Congenital heart disease

## Abstract

**Background:**

Systemic right ventricle (RV) hypertrophy and impaired function occur after atrial switch for dextro-transposition of the great arteries (d-TGA). Echocardiography is limited in its ability to assess the RV. We sought to evaluate systemic RV myocardial-mass index (MMI) and function after atrial switch and to analyse the role of hypertrophy for ventricular function with special consideration of the interventricular septal (IVS) movement.

**Methods:**

Thirty-seven consecutive patients (median age 22.9 years) after atrial switch were studied using cardiac magnetic resonance imaging (1.5T Intera, Philips) with a dedicated 5-channel phased-array surface cardiac coil. Cine steady-state free-precession sequences were acquired to obtain myocardial masses and function. The systolic movement of the IVS was defined as positive when moving towards the centroid of the RV and was defined as non-positive otherwise. Patient parameters were compared to controls.

**Results:**

The systemic RVs were significantly larger (*p* < 0.001) than the left ventricles of the control group, systolic function was significantly impaired (*p* < 0.001) and MMI including the IVS was comparable (*p* = n.s.). RV-MMI excluding the IVS and RV ejection fraction (EF) demonstrated a quadratic correlation (*r* = 0.6, *p* < 0.001), meaning that patients with RV-MMI ≤29 g/m^2^ and >68 g/m^2^ had a reduced level of systolic function. Positive septal movement improved RV function compared with non-positive septal movement (*p* = 0.024).

**Conclusions:**

There seems to be a range of beneficial RV hypertrophy after atrial switch in which a sufficient RV-EF can be expected. A positive septal movement, probably the result of hypertrophic septal RV fibres, improves RV function and might be regarded as a beneficial contraction pattern.

## Background

Dextro-transposition of the great arteries (d-TGA) is one of the most common severe cardiac lesions [1]. Before anatomical correction became the procedure of choice for surgical treatment of d-TGA, the atrial switch operation was performed to achieve a physiological correction of blood flow [2, 3]. The midterm and long-term benefits of this procedure are moderate, and there are specific long-term complications that are associated with increased morbidity and mortality [4]. In addition to the development of arrhythmias, systemic ventricular dysfunction is the most important sequela, being mainly caused by the non-physiological systemic position of the right ventricle (RV) [5]. Treatment of RV dysfunction is challenging, due to chronic pressure overload and structural myocardial remodelling in the form of myocardial fibrosis. Therefore, to optimise therapeutic strategies, an early and precise diagnosis is mandatory.

Today, there are many patients who are in need of regular follow-up assessment of cardiac function. However, assessment of RV dimensions and function by echocardiography is difficult, due to the retrosternal position and complex chamber shape of the RV. It has been shown, that compared with cardiac magnetic resonance (CMR), echocardiography underestimates systemic RV function, and has a constantly lower image quality and an impaired capability to visualise the baffles [6, 7]. Therefore, CMR imaging has been established as a powerful tool for the assessment of biventricular morphology and function in patients after atrial switch operation [8, 9].

Some studies have analysed the specific changes associated with the function of the RV as the systemic ventricle [10, 11], but the reasons for RV failure are still controversial. Hornung et al. [12] have identified excessive RV hypertrophy as a potential risk factor, but, to date, it is unclear which degree of hypertrophy is beneficial for systemic RV function.

The aim of this study was to analyse the role of RV hypertrophy in patients following atrial switch for d-TGA and to acquire volumetric and functional values as a backup for therapeutic monitoring.

## Methods

### Patient population, control group and study design

This is a retrospective study. Thirty-seven consecutive patients who were regular outpatients at our tertiary care institution were referred for CMR. The eligibility criteria were an atrial switch operation in childhood for correction of d-TGA and the absence of associated haemodynamically significant heart defects. The exclusion criteria consisted of the usual CMR contraindications, such as ferromagnetic metallic implants like defibrillators. Patients underwent echocardiography by experienced cardiologists within 2 days of the CMR. Tricuspid insufficiency (TI) was categorised as absent, mild, moderate or severe according to the current guidelines of the European Society of Cardiology [13]. Baffle obstruction by Doppler echocardiography was defined as a pulse-wave Doppler peak velocity of ≥1.5 m/s [14]. Pulmonary stenosis was either defined as a peak velocity of >3.2 m/s in Doppler echocardiography or a pressure gradient of >40 mmHg in cardiac catheterization [15]. New York Heart Association (NYHA) functional class was assessed by patient interrogation. If available, the peak oxygen uptake (*V*O_2max_) from spiroergometry [16] was obtained. Patient parameters were compared to a control group of 19 healthy volunteers. The study was approved by the local ethics committee and complied with the Declaration of Helsinki. All patients or parents gave written informed consent.

### CMR imaging

All CMR examinations were performed on a 1.5 T scanner (Intera CV, Philips Medical Systems, Best, The Netherlands) using a dedicated 5-channel phased-array surface cardiac coil. For volumetric and functional imaging, breath-hold standard cine steady-state free-precession sequences in short-axis, 4-chamber view and RV vertical long-axis orientation were acquired. Short-axis images covered the whole heart gapless from the apex to the base (30 phases per heart cycle). The following sequence parameters were used: echo time, 1.8 ms; repetition time, 3.6 ms; flip angle, 50°; matrix size, 256 × 128; and slice thickness, 8 mm.

CMR image analysis was performed by two fully blinded observers (M.G. and J.H.) with 13 and 5 years of experience in CMR, respectively, using a Philips ViewForum workstation (Version 4.2; Cardiac Evaluation Package) in our CMR laboratory, which has demonstrated expertise in the imaging of congenital heart disease and has proven low intra- and inter-observer variability in the assessment of biventricular volumes and function [17–19]. Endocardial and epicardial borders were manually traced during end systole and end diastole in each slice. Trabeculation was excluded from calculation of myocardial mass and included in the ventricular cavity. A narrowing of the systemic venous baffle was defined as a transverse luminal diameter of <10 mm as described before [14].

The following parameters were calculated for the RV and left ventricle (LV) and were related to body surface area [20]:

- End-diastolic volume index (EDVI)
- End-systolic volume index (ESVI)
- Stroke volume index
- Myocardial mass index (MMI)
- Ejection fraction (EF).

**Fig. 1:**
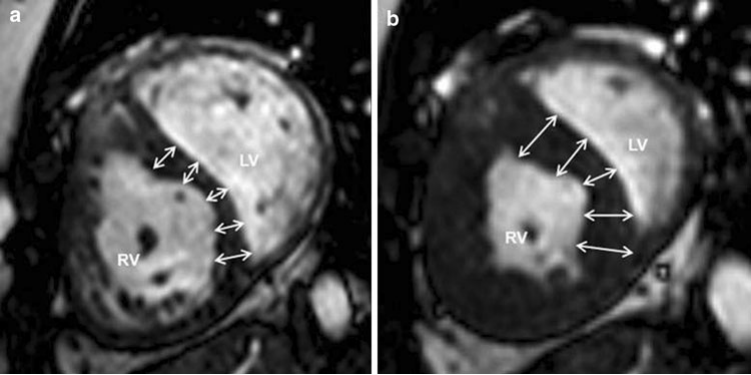
Positive septal movement in short-axis cine SSFP images. Cine steady-state-free-precession (SSFP) images in a short-axis orientation. In diastole (**a**) the interventricular septum (IVS) is thin. In systole (**b**) the IVS shows a substantial wall thickening and a movement towards the free wall of the right ventricle. This active contraction pattern was defined as positive septal movement

For calculation of the myocardial mass, a specific density of 1.05 g/ml was used. The mass of the interventricular septum (IVS) was assessed separately. The movement of the IVS was classified as positive or non-positive. A positive septal movement was defined as a systolic movement of the septum toward the centroid of the RV cavity and systolic wall thickening (Fig. 1), indicating an active process in contrast to a paradoxical septal movement without wall thickening [21]. The non-positive septal movement included all other forms of septal movement, including either a systolic movement towards the lateral wall of the LV or a movement of the IVS towards the RV free wall without systolic wall thickening (paradoxical septal movement).

### Statistics

Owing to the heterogeneity of the data in Levene’s test, the relatively small patient cohort and the non-normal distribution for most parameters in the Kolmogorov–Smirnov test, continuous data were expressed as medians [25 %; 75 % percentiles]. The nonparametric Mann–Whitney *U* test was performed to compare volumetric measurements and differences in the demographic parameters. For the analysis of binomial parameters, we used the Chi-square test. The scatterplot of RV-EF versus RV-MMI reveals a nonlinear behaviour. Fitting a quadratic in place of a linear regression model improved the coefficient of determination. Statistically important associations between different volumes were examined using linear and quadratic regression analyses. Differences between variables were demonstrated using box-and-whisker plots, and regression analysis results were presented using scatter plots.

The analyses were performed using 2-sided tests with a significance level of *p* = 0.05. SPSS software version 16.0 (SPSS Inc., Chicago, Il, USA) was used.

## Results

The length of the CMR imaging was ≤1 h in all 37 patients and 19 controls, and diagnostic image quality was obtained. The patient and control group characteristics are presented in Table 1. The distributions of the patient and control group volumetric and functional parameters are summarised in Table 2.

**Table 1:** Patient and control group characteristics

	Patient group (*n* = 37)	Control group (*n* = 19)
Sex male/female	25 (69 %)/12 (32 %)	12 (63 %)/7 (27 %)
Body surface area (m^2^)	1.80 [1.59;2.00]	1.82 [1.71;2.00]
Age at examination (years)	22.9 [18.0;26.9]	23.3 [20.6;23.9]
Age at surgical correction (months)	15.0 [7.0;22.8]	–
Postoperative interval (years)	21.5 [17.5;24.9]	–
Mustard/Senning	15 (41 %)/22 (59 %)	–

Continuous data are presented as median [interquartile range]. Categorical data are presented as frequency and percentage

**Table 2:** Patient and control group parameters

Parameters	Patient group (*n* = 37)	Control group (*n* = 19)	*p*
RV end-diastolic volume index (ml/m^2^)	92 [79;117]	75 [69;85]	<0.001
RV end-systolic volume index (ml/m^2^)	47 [38;73]	32 [28;36]	<0.001
RV stroke volume index (ml/m^2^)	44 [35;53]	43 [39;54]	0.28
RV ejection fraction ( %)	44 [36;53]	57 [55;63]	<0.001
RV myocardial mass index			
Without IVS mass (g/m^2^)	41 [34;48]	15 [13;17]	<0.001
With IVS mass (g/m^2^)	54 [44;61]	30 [28;32]	<0.001
LV end-diastolic volume index (ml/m^2^)	60 [49;76]	78 [71;86]	0.01
LV end-systolic volume index (ml/m^2^)	27 [18, 35]	27 [24;29]	0.79
LV stroke volume index (ml/m^2^)	35 [30;44]	51 [47;56]	<0.001
LV ejection fraction ( %)	58 [51;67]	65 [62;67]	0.01
LV myocardial mass index			
Without IVS mass (g/m^2^)	16 [11, 21]	41 [35;50]	<0.001
With IVS mass (g/m^2^)	30 [22;64]	52 [49;68]	<0.001
IVS myocardial mass index (g/m^2^)	11 [9;15]	16 [13;18]	0.01
Total myocardial mass index (g/m^2^)	71 [60;82]	68 [65;81]	0.98
NYHA functional class			
I	24	19	
II	12	0	
III	1	0	
IV	0	0	
Peak oxygen uptake (ml/kg/min), *n* = 27	25 [17;32]		
Tricuspid insufficiency			
No	10	19	
Mild	18	0	
Moderate	9	0	
Severe	0	0	

Data are presented as median [interquartile range]

*RV* right ventricle, *IVS* interventricular septum, *LV* left ventricle, *NYHA* New York Heart Association

### Baffle and tricuspid function

All patients presented with typical postoperative morphology, which included a large dilated hypertrophic RV, a small hypotrophic LV and a systemic venous and pulmonary venous baffle. There were two systemic venous baffle narrowings of the superior limb with a diameter of <10 mm. None of these patients showed a pulse-wave Doppler peak velocity of ≥1.5 m/s. These baffle narrowings were therefore regarded as not hemodynamically significant. Seven patients demonstrated a peak velocity of >3.2 m/s in the pulmonary trunk. In two of these patients a cardiac catheterization was also performed and the diagnosis of a pulmonary stenosis with pressure gradients of above 40 mmHg was confirmed. Of the seven patients with a pulmonary stenosis, two had a positive and five a non-positive septal movement. This difference was not statistically significant (*p* = 0.58).

Echocardiography showed that 10 patients had nonexistent, 18 had mild and 9 had moderate TI. There were no differences between the patients without TI and patients with TI regarding RV-EDVI (*p* = 0.58), RV-ESVI (*p* = 0.81) and RV-EF (*p* = 0.92).

### RV-EF, clinical, functional and volumetric parameters

The systemic RV-EF showed different grades of systolic function (Table 2); however, the majority of patients (*n* = 23) presented with relatively sufficient RV-EFs of >40 % according to the guidelines of the European Society of Cardiology [22]. The RV-EF was not related to the subjectively reported physical performance of the patients. Out of 37 patients, 24 (65 %) were in good clinical condition and were graded as NYHA functional Class 1, 12 (32 %) were Class 2 and only 1 (3 %) was Class 3. None of our patients was Class 4. Due to the retrospective character of this study, *V*O_2_ values from spiroergometry were only available in 27 patients (Table 2). There was no linear or quadratic correlation between these values and RV-MMI, but a weak positive linear correlation of *V*O_2max_ values and RV-EFs (*r* = 0.35, *p* = 0.10).

**Fig. 2:**
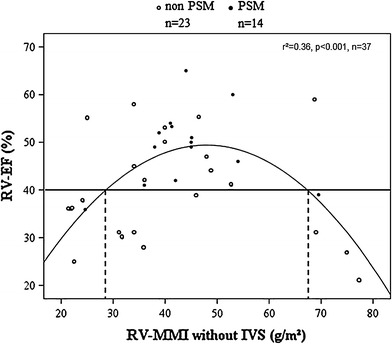
Correlation between right ventricular hypertrophy, function and IVS movement. *Scatterplot* diagram demonstrating a quadratic regression of right ventricular myocardial mass index (*RV MMI*) and RV ejection fraction (*RV EF*). There is impaired RV-EF with very low and very high RV-MMI. Most patients with a RV-MMI within the beneficial range have a positive septal movement, whereas patients with a very low and a very high RV-MMI have a non-positive septal movement in most cases. *IVS* interventricular septum, *PSM* positive septal movement

The fit of a quadratic model for the nonlinear relationship between the RV-EF and RV-MMI excluding the IVS (RV-EF = −0.025 × RV-MMI^2^ − 2.404 × RV-MMI − 8.25) is acceptably well (*r* ^2^ = 0.36, *p* < 0.001). It explains 36 % of the variance. In this context *r* ^2^ can be regarded as goodness of fit. The lower threshold for a RV-EF >40 % was a RV-MMI excluding the IVS of 29 g/m^2^, the upper threshold a RV-MMI excluding the septum of 68 g/m^2^ (Fig. 2), meaning that lower MMIs showed a positive correlation and higher MMIs showed a negative correlation with the ventricular function expressed as the RV-EF. Furthermore, RV-MMI excluding the septum was positively correlated with the RV-EDVI (*r* = 0.52, *p* = 0.001) and with the RV-ESVI (*r* = 0.33, *p* = 0.04). There was no significant difference in RV-EDVI (*p* = 0.65) and RV-ESVI (*p* = 0.33) in patients with a RV-EF of <40 % and RV-MMI of <29 g/m^2^ as compared to the remaining patients. There was also no difference in RV-EDVI (*p* = 0.21) and RV-ESVI (*p* = 0.20) in patients with a RV-EF <40 % and RV-MMI <29 g/m^2^ as compared to patients with a RV-EF <40 % and a RV-MMI >68 g/m^2^. However, when the latter two groups were combined to one group, there was a significantly higher RV-ESVI as compared to the remaining patients (75.4 [47.0;113.5] ml/m^2^ vs. 46.0 [37.0;63.7] ml/m^2^, *p* = 0.04). There was a negative correlation of RV-EF and RV-EDVI (*r* = −0.46, *p* = 0.05) and a negative correlation of RV-EF and RV-ESVI (*r* = −0.69, *p* < 0.001).

**Fig. 3:**
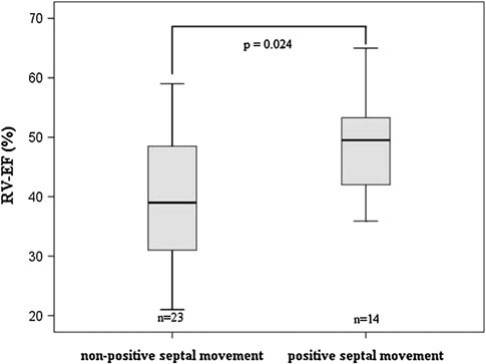
Positive septal movement and right ventricular function. *Boxplot* diagram for comparison of systemic right ventricular ejection fraction (*RV EF*) grouped according to the presence or absence of a positive septal movement. Patients with positive septal movement show a significantly better systolic function

Fourteen patients had a positive septal movement. The systemic RV-EF was substantially higher in the positive septal movement patients (49 %) than in the non-positive septal movement patients (37 %, *p* = 0.024, Fig. 3). The IVS-MMI was not significantly higher in patients with a positive septal movement than in patients without a positive septal movement (13.0 [10.5;16.6] g/m^2^ vs. 10.8 [9.0;16.2] g/m^2^, *p* = 0.53). No differences were found in the RV-MMI excluding the IVS, LV-EF and LV-MMI between the positive septal movement patients and the non-positive septal movement patients.

There was a weak correlation between the RV-MMI excluding the IVS and the duration of the postoperative interval (*r* = 0.39, *p* = 0.02). There was a weak correlation of the duration of the postoperative interval and the RV-EF (*r* = 0.32, *p* = 0.09). RV-EDVI and RV-ESVI did not correlate with the postoperative interval. Age at atrial repair did not correlate with either RV-MMI or RV-EF. There was no gender predominance for RV hypertrophy.

### Systemic RVs compared with systemic LVs and pulmonary RVs

Systemic RVs of the patient group were significantly larger compared with systemic LVs of the control group (92 [79;117] ml/m^2^ vs. 78 [71;86] ml/m^2^, *p* = 0.01) and the global systolic function was significantly impaired (44 [36;53] % vs. 65 [62;67] %, *p* < 0.001). MMI showed no significant differences between patients and controls, irrespective of whether the IVS was excluded (41 [35;50] g/m^2^ vs. 41 [34;49] g/m^2^) or included (54 [44;61] g/m^2^ vs. 52 [49;68] b g/m^2^) to the systemic ventricles.

Compared with the pulmonary RVs, the MMI of the systemic RVs was almost twice as high when the IVS was included and almost three times as high when the IVS was excluded from the comparison. The total IVS-MMI of the patients did not differ significantly from the control group (Table 2).

## Discussion

This study is one of the largest CMR studies to measure cardiac volumes, myocardial mass and function in patients after the atrial switch procedure for d-TGA and to measure the procedure’s effects on the systemic function. The main purpose was the analysis and characterisation of myocardial hypertrophy of the RV in the systemic position. We demonstrated remodelling of the myocardial mass in patients after atrial switch. Our results can be summarised as follows:

- There seems to be a range of beneficial hypertrophy of the systemic RV, which is mainly a hypertrophy of the free wall of the RV and its trabecular parts.
- Positive septal movement supports systemic RV function without impairing LV function, which is probably caused by disproportionate hypertrophy of the septal RV fibres.

### Systemic systolic function and the role of RV hypertrophy

The majority of our patients showed a RV-EF >40 % and a good self-reported functional status, even after a mean follow-up period of 21.5 years. This is in line with the findings of a recently published work with a comparable follow-up period of 19.5 years [23]. Here, 12 % of patients had a RV-EF <40 % and clinically significant impairment was only present in one patient. In a further study, analysing 35 d-TGA patients after atrial switch, mean RV-EF was >50 % and all patients were NYHA Class I or II [24].

None of the cited studies analysed RV-MMI, yet RV hypertrophy is a central mechanism in the adaptation process after atrial switch operation. The RV is constructed as a volume pump with macroscopic and microscopic differences compared with the LV pressure pump in terms of trabecular and fibral architecture [25, 26]. To date, the role of RV hypertrophy is not fully understood. Hornung et al. [12] demonstrated an inverse correlation of the RV-MMI and RV-EF, thereby interpreting hypertrophy as a harmful process in atrial switch circulation.

In the present work RV hypertrophy showed a range within which systemic RVs demonstrated an acceptable systolic function. The initially beneficial process of hypertrophy probably becomes detrimental when excessive and presumably chronic ischaemia with consecutive fibrosis of the RV occurs. Recent late enhancement studies showed that the presence and extent of myocardial fibrosis correlates with impaired systolic function and suggested that RV fibrosis ensues when a certain threshold of RV mass is reached [27, 28]. Our results support the concept of an upper threshold but, additionally, introduce a lower threshold of RV hypertrophy because patients with a low hypertrophic response to pressure load also presented with systemic dysfunction. The effects of TI on RV-EF could be excluded.

Although our patient cohort was relatively homogeneous with regard to the age and method of surgical repair, it is not possible to define general concrete upper and lower cut-off values for the RV-MMI, given that patients after atrial switch as a whole are a heterogeneous group. Nevertheless, our cut-off values of 29–68 g/m^2^ may serve as an orientation, and it may be highly interesting if these values are comparable for other patients with right systemic RVs without palliative operations (e.g., in congenitally corrected TGA).

**Fig. 4:**
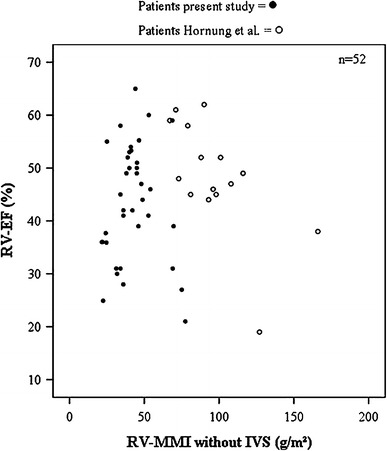
Distribution of RV mass and function. *Scatterplot* diagram showing the right ventricular myocardial mass index (*RV-MMI*) and the RV ejection fraction (*RV-EF*) of patients of the present study and the patients of Hornung et al. [16]. There is a parallelly shifted decline of RV systolic function in both patient groups. Patients of the cited study have higher calculated RV-MMIs

The study of Hornung et al. [12], with a similar postoperative interval, found a higher mean RV-MMI after atrial switch compared to the present study. The authors used a threshold of 95 g/m^2^, above which RV function was impaired compared to patients with a RV-MMI below 95 g/m^2^. Figure 4 shows RV mass and RV function of our patients and the patients of Hornung et al. in one scatterplot diagram. There are two major issues that might explain the discrepancy in RV mass. First, the age at atrial switch procedure was lower in our patient cohort, which might have resulted in a different adaptation to systemic pressure load. Second, there was no detailed description of the assessment of the RV mass in the cited study with regard to exclusion or inclusion of RV trabeculation. In the present study, we traced endocardial borders manually and excluded RV trabeculation from calculation of RV-MMI, which is a standard procedure in ventricular volumetry and has shown better reproducibility in systemic RVs [29]. An automated threshold-based segmentation, including trabeculated myocardium, might provide more precise values.

In our study, the patients’ RV-MMI showed a wide distribution of measured values. There was no single parameter that clearly correlated with inadequate or excessive hypertrophy, but a weak correlation with the duration of the postoperative interval was found. Furthermore, there was a weak dependency of systemic RV function on the duration of the postoperative interval. A previously published non-CMR study has also demonstrated impaired systemic RV function in the late follow-up after atrial switch [30]. The time factor might be one parameter in a multifactorial process.

Patients with an inadequate hypertrophic or excessive response to pressure load and an impaired RV function showed increased RV-ESVIs, which were also found in the subgroup of patients with a non-positive septal movement. These changes might reflect abnormal RV dilatation in the absence of TI. An increase of the end-systolic volume is a marker of remodelling and the most frequent cause of impaired EF in systemic LVs. Also in the present study RV-ESVI was the parameter with the strongest correlation to systolic function. We conclude that remodelling is also present in systemic RVs after atrial switch and results in impaired function.

There was no correlation between the degree of systemic RV hypertrophy and exercise tolerance tests and only a tendency of higher *V*O_2max_ values with higher RV-EFs. In our opinion, these results do not contradict our findings. The lack of correlation between exercise capacity and ejection fraction in rest is a well-known finding in systemic LVs [31] and might also apply for systemic RVs. Furthermore, owing to the retrospective character of this study, spiroergometry was performed up to 2 years from the date of the CMR examination.

We also did not find a male predominance in RV hypertrophy as has been described previously [12]. It remains unclear what additional factors might influence the degree of RV hypertrophy. Different intensities of the various molecular response mechanisms that control myocardial hypertrophy probably play an important role both in normal LVs and in RVs coping with sustained increased blood pressure [32].

### Role of the IVS

We defined positive septal movement as an active movement of the IVS toward the RV free wall, which is indicated by a systolic wall thickening. More than a third of our patients presented with positive septal movement, which was found beneficial for the calculated RV-EF without impairing global LV-EF. Positive septal movement might, therefore, be regarded as a beneficial contraction pattern after atrial switch because it supports systemic RV function without impairing LV function, which is probably caused by disproportionate hypertrophy of the septal RV fibres. Patients with a positive septal movement showed a slightly higher IVS-MMI compared with patients without a positive septal movement. The missing statistical difference between these two subgroups might again be caused by the small sample size. It is also possible, that the underlying mechanism of a positive septal movement is multifactorial an even a marginal increase of RV-MMI could be the determining factor. The distribution of RV-MMI excluding the septum shows, that the presence of a positive septal movement is associated with adequate hypertrophic response in most cases (Fig. 2). Therefore, it might be possible that there is an interaction between the RV-MMI without the septum and the MMI of the RV septal portion that cannot be measured in the present study.

Furthermore, the septum consists of RV and LV fibres. If the hypertrophy of RV fibres is not much more than the LV fibres’ hypotrophy due to the adaptational process, the measurement of the absolute volumes of the septal muscle mass by MRI cannot reveal this effect and may mask the septal RV fibres’ hypertrophy.

Nevertheless, further larger studies will have to prove or disprove our assumption of a disproportionate hypertrophy of the septal RV fibres.

The pattern of IVS movement was not dependent on the presence or absence of pulmonary stenosis. This is contradictory to the results of a previous study that reported a commitment of the IVS to the LV in the presence of a pulmonary stenosis [33]. One reason for this discrepancy might be differences in the study cohorts. The cited study also included 21 patients with a congenitally corrected TGA, who have a different physiology of the systemic right ventricle compared with patients with d-TGA after atrial switch.

LV-MMI was not different between the patients with positive and non-positive septal movement, thereby emphasising that positive septal movement is not simply the absence of LV hypertrophy (e.g., in pulmonary hypertension). Since the myocardial septum is a structural part of both the RV and the LV, remodelling in the LV portion may compensate or even exceed the hypertrophy of the RV portion, resulting in equal or impaired IVS-MMI compared with that of the controls. However, the RV portion of the IVS is difficult to obtain separately in standard CMR because it contains a thin compacted layer and many trabeculations [34]. Modern techniques such as CMR diffusion tensor imaging will provide deeper insights into the myocardial fibre architecture of the IVS and the phenomenon of positive septal movement in patients after atrial switch [35].

### Systemic RV compared with systemic LV and pulmonary RV

Systemic RV size was markedly increased compared with both RV and LV size of the controls. Dilatation of the systemic RV is a common finding after atrial switch [6, 11] and is regarded as a result of TI and the incompetence of the right coronary artery to provide sufficient oxygen supply of the hypertrophic myocardium, thereby resulting in remodelling [36]. Our results support this hypothesis by demonstrating that the progression of RV dilatation was also associated with increased RV mass. Additionally, although the systemic RV mass was not different from the systemic LV mass of the controls, the RV-EF was lower. This finding is to be expected, because the RV is genetically disposed to pump blood at lower pressure to the pulmonary circulation. The adaptation in the systemic position occurs in the form of dilatation and increased myocardial mass. We speculate that dilatation and the abnormal specifics of the RV myocardial architecture led to an inefficient systolic function in the systemic RV.

### Limitations

This is a retrospective study. Exercise testing was only available in 27/37 patients, which limits the statistical power of this analysis. The NYHA classification has limitations because self-reported ability sometimes does not match the result of standardised exercise capacity. We did not perform a systematic evaluation of NT-pro-BNP levels, although the emerging importance of biomarkers in the diagnosis of heart failure has been described in recent studies [37, 38].

Patients with a manifest ventricular tachycardia and an implanted cardioverter/defibrillator had to be excluded from the study. Thus, it was not possible to correlate the obtained parameters with an interesting subgroup of patients. Since most patients after atrial switch are in good clinical condition over a long period of time, “hard” clinical endpoints, such as death or congestive heart failure, are rare and can only be used in large multicentre studies. Furthermore, we did not perform CMR delayed enhancement imaging to measure systemic RV myocardial fibrosis.

## Conclusions

Systemic RV hypertrophy after atrial switch procedure in d-TGA is a physiologic adaptational process that allows for building up systemic pressure. Excessive RV hypertrophy, however, can result in impaired systolic function as described before. Our results support the concept of an upper threshold but, additionally, introduce a lower threshold of RV hypertrophy, because patients with a low hypertrophic response to pressure load also presented with systemic dysfunction. It remains unclear though, which patients are at risk of this inadequate response.

An active movement of the IVS towards the RV cavity, which is probably caused by disproportionate hypertrophy of the septal RV fibres, supports systemic RV function without impairing LV function and might therefore be regarded as a beneficial contraction pattern after atrial switch procedure.
